# Angiographic Imaging of Prostatic Artery Origin in a Greek Population and Correlation With Technical and Clinical Aspects of Prostatic Artery Embolization

**DOI:** 10.7759/cureus.45941

**Published:** 2023-09-25

**Authors:** Hippocrates Moschouris, Konstantinos Stamatiou, Spyros Tzamarias, Konstantinos Frigkas, Nektarios Spanomanolis, Ivlitta Isaakidou, Effrosyni Dimitroula, Stavros Spiliopoulos, Elias Brountzos, Katerina Malagari

**Affiliations:** 1 Radiology, Tzanio General Hospital, Piraeus, GRC; 2 Urology, Tzanio General Hospital, Piraeus, GRC; 3 Radiology, University Hospital of Alexandroupolis, Alexandroupolis, GRC; 4 Second Department of Radiology, Attikon University Hospital, Athens, GRC

**Keywords:** dose area product, fluoroscopy time, catheterization, computed tomographic angiography, digital subtraction angiography, prostatic artery anatomy, prostatic artery origin

## Abstract

Background

This study aimed, first, to angiographically investigate and analyze prostatic artery (PA) origin in a Greek male population with benign prostatic hyperplasia (BPH) treated with prostatic artery embolization (PAE) and, second, to correlate prostatic arterial anatomy with technical and clinical aspects of PAE.

Methodology

This was a retrospective study of BPH patients who underwent PAE in a single tertiary center in Greece from June 2019 to July 2022. For the first part of the study, PA was imaged with computed tomography angiography (CTA) before PAE and with digital subtraction angiography (DSA) during PAE in all patients. A widely accepted system for the classification of PA origin was applied. Type I, a common origin of PA and superior vesical artery (SVA) from the anterior division of internal iliac artery (IIA). Type II, PA originating from the anterior division of IIA, separate from, and inferior to SVA. Type III, the origin of PA from the obturator artery. Type IV, the origin of PA from the internal pudendal artery. Type V, rarer origins of PA. For the second part of the study, a subgroup of patients from the first part (treated with the same PAE protocol and free of vascular pathology that could have interfered with the technical success of PAE) was selected. In this subgroup, differences in PA origin were correlated with technical aspects (feasibility of catheterization of PA, fluoroscopy time (FT), dose area product (DAP)) and clinical outcomes of PAE.

Results

After the exclusion of four patients, 159 patients were included in the first part of the study. From a total of 355 PAs, 110 (31%) were compatible with type I, 58 (16.3%) type II, 45 (12.7%) type III, 110 (31%) type IV, and 32 (9%) type V. PA origin from an accessory internal pudendal artery was the most common among the rare origins of type V. Regarding the second part of the study (a subgroup of 101 patients selected to facilitate comparisons between the different types of PA origin), type I was associated with significantly more incidences of failed or difficult catheterization of the PA compared to all other types combined (27/64 vs. 18/138, p < 0.001). Types III, IV, and V showed a relatively low degree of technical difficulty. Patients with type I PA origin of at least one pelvic side (subgroup (I), n = 48) had significantly longer FT and DAP compared to the rest (subgroup (O), n = 53). Clinical success rates of PAE were slightly lower for the subgroup (I), although the difference was not statistically significant (75.8% vs. 83.8% at 18 months post-PAE, p = 0.137). No major complications were observed.

Conclusions

This is the first study of PA origin in Greece. It was demonstrated that types I and IV of PA origin were the most common and had the same prevalence. Type I showed significantly higher technical difficulty compared to the others, but had no significant impact on the clinical outcomes of PAE.

## Introduction

During the last 10 years, imaging of prostatic artery (PA) anatomy has been attracting researchers’ interest as a result of the increasing application and promising outcomes of prostatic artery embolization (PAE) for the treatment of symptomatic benign prostatic hyperplasia (BPH) [1]. Among other anatomic features of PA, the site of PA origin shows considerable variation and probably plays a role in the technical outcomes of PAE [2]. In 2015, de Assis et al. [3], described a practical and clinically useful angiographic classification of PA origin as follows: Type I, common trunk of PA with the superior vesical artery. In type II, PA originates from the anterior division of the internal iliac artery (IIA). In type III, PA originates from the obturator artery. In type IV, PA originates from the internal pudendal artery (IPA). Type V includes rare PA origins. Subsequent studies [4-6] from different countries utilizing this classification system revealed remarkable variations in the prevalence of the aforementioned types of PA origin. There is probably a place for further research in this field, including populations from countries other than the already studied to enhance knowledge on the variations of prostatic arterial anatomy worldwide.

Furthermore, the correlation of different types of PA origin with certain technical or clinical aspects of PAE could facilitate preparation for PAE, treatment planning, and patient counseling. If, for example, pre-interventional imaging reveals a ‘’difficult’’ type of PA origin, on-site availability of more dedicated devices (such as special microcatheters or microguidewires) should be ensured, and the patient should also be informed of the possibility of a lengthy or unsuccessful procedure.

Taking into account the paucity of relevant data from the Balkan region (and particularly from the country of the authors), a study was conducted to study the prevalence of the various types of PA origin in a Greek population treated with PAE for BPH, as well as to investigate if the type of PA origin has any impact on technical aspects and clinical outcomes of PAE.

## Materials and methods

A retrospective, single-center study of a Greek male population treated with PAE was conducted. Inclusion criteria were similar to those of previous work [7] and included age >50 years; moderate-to-severe lower urinary tract symptoms (International Prostate Symptom Score, IPSS >18) or hematuria as a result of BPH; failure of medical treatment or urinary retention managed with indwelling bladder catheter (IBC); and prostate volume higher than 40 mL. Exclusion criteria were urinary tract infection, prostate or bladder cancer, neurogenic bladder, large (>3 cm) bladder diverticula or bladder stones, and angiographic contraindications. Written informed consent was obtained from all patients before PAE. The study was approved by the institutional review board (approval number: 11272/2023).

For the first part of the study, angiographic imaging of PA origin was based on two imaging modalities. The first was computed tomographic angiography. This was performed before PAE, with a 64-row scanner (Optima CT 660, GE Healthcare, WI, USA) using the following scanning parameters: 120 kV, automatic milliamperage, matrix of 512 x 512 pixels, collimation of 64 x 0.625 mm, slice thickness of 1.25 mm, and pitch of 0.984:1. Iodinated contrast (1.5 mL/kg of patient’s weight, 350 mg iodine/mL) was injected with mechanical injector in a forearm vein at a rate of 4.5-5 mL/second. For bolus triggering, a region of interest was placed just above aortic bifurcation, and scanning started when a threshold of 250 HU was reached. Vasodilatation before CTA was achieved using a sublingual spray of glyceryl trinitrate (2 x 400 µg).

The course of the PAs was first studied on thin (0.625 mm) axial CT slices and PAs were tracked from the prostate capsule back to their origin from the parent artery. Subsequently, maximum intensity projection (MIP) images were produced on coronal, sagittal, and various oblique sagittal planes. MIPs that most clearly showed PA origins were selected for the angiographic analysis and for correlation with DSA images.

The second imaging modality was digital subtraction angiography (DSA). This was performed during PAE with a flat-panel angiographic unit (Axiom Artis Zee, Siemens Healthineers, Erlangen, Germany). After catheterization of the IIAs with a 5 French (Fr.) angiographic catheter, DSA was performed with manual injection of 10-20 mL of iodinated contrast (300 mg iodine/mL) at a rate of 1 frame/second with the appropriate (‘’Body CARE 1’’) angiographic protocol. The X-ray tube angulation was adjusted according to the obliquity of the aforementioned MIP images that most clearly showed PA origin. Most commonly, DSA was performed in ipsilateral oblique (30-40°) projection with occasional additional slight (5°) caudocranial angulation. Additional DSA runs at other projections were acquired if deemed necessary.

DSA and CTA/MIP images were reviewed and correlated by two radiologists (each with seven years of experience in PAE) and the number and type of PA origin(s) were defined on each pelvic side. Cases of discordance between DSA and CTA/MIP images that were encountered during this review process were resolved by consensus between the two reviewers, otherwise, they were excluded. Also excluded were cases in which no PA could be identified on the pelvic side by one or both of the imaging modalities. DSA runs after super-selective cannulation of PA with a microcatheter were also reviewed; however, they were not considered mandatory for the characterization of PA origin. Cone-beam computed tomography (CBCT) was also optional and only occasionally performed in the context of this study.

For the second part of the study, a subgroup of patients was selected who had undergone PAE with the same technique and devices (Table 1) and were free from vascular pathology and other factors that could have interfered with the technical success of PAE. For each of these patients, the PAE procedure was retrospectively reviewed in detail (including written reports regarding the utilized devices and procedural steps, static angiographic images, and videofluoroscopic acquisitions showing the catheterization and embolization procedure). Super-selective catheterization of PA was characterized as (1) ‘’Successful’, if it could be accomplished with the standard, initially utilized combination of microcatheter/microguidewire; (2) ‘’Difficult’’ if super-selective PA catheterization required utilization of additional dedicated microcatheter/microguidewire; and (3) ‘’Failed’’ if super-selective PA catheterization could not be accomplished despite the utilization of both standard and additional dedicated devices. Radiation data for each PAE procedure (fluoroscopy time (FT) and dose area product (DAP)) were also collected from the angiographic unit and correlated with differences in PA origin. Finally, the subgroup of patients for the second part of the study was followed up and the clinical outcomes as well as the safety of PAE were evaluated. The following widely accepted criteria of clinical success of PAE [7] were applied: (1) Post-PAE International Prostate Symptom Score (IPSS) ≤15 points with a decrease of at least 25% from the baseline; (2) Post-PAE quality of life (QoL) score ≤3 points or a decrease of at least 1 point from baseline; (3) No requirement for medical or surgical treatment post-PAE. For patients with chronic urinary retention and indwelling bladder catheter, clinical success included successful and permanent catheter removal with spontaneous micturition and post-void residual <100 mL. Successful PAE for hematuria of prostatic origin was defined as complete cessation of bleeding post-PAE.

**Table 1:** Basic features of the PAE procedure applied among the patients (n = 101) during the second part of the study. Fr: French; IIA: internal iliac artery; PA: prostatic artery

Feature	Details
Access	From right or left common femoral artery (5 Fr vascular sheath)
Catheterization of IIA	Ipsilaterally with 5 Fr, reverse-curve angiographic catheter. Contralaterally, after cross-over maneuver and with the previously used catheter or a vertebral-type catheter
Super-selective catheterization of PA: a standard combination of devices	2.0 Fr microcatheter (Parkway soft Asahi Intecc, Japan) 0.0016" microguidewire (Meister Double-Angle, Asahi Intecc, Japan)
Super-selective catheterization of PA: devices used if standard combination failed	Steerable microcatheter (SwiftNinja, Merit Medical, USA) ± additional microguidewires
Embolization material	Microspheres, diameter of 100–300 µ (Embosphere, Merit Medical, USA)

Descriptive statistics were calculated for quantitative and qualitative data. The normality of data distribution was assessed using the Shapiro-Wilk test. Independent-sample t-test was used for the comparison of means between subgroups. The chi-square test was used for the comparison of proportions (for example, technical failure rates between different types of PA origin). The Kaplan-Meier method was used to calculate clinical success rates of PAE over time and the log-rank test to compare differences in clinical success rates between subgroups. Statistical significance was defined as a p-value <0.05.

## Results

**Figure 1:**
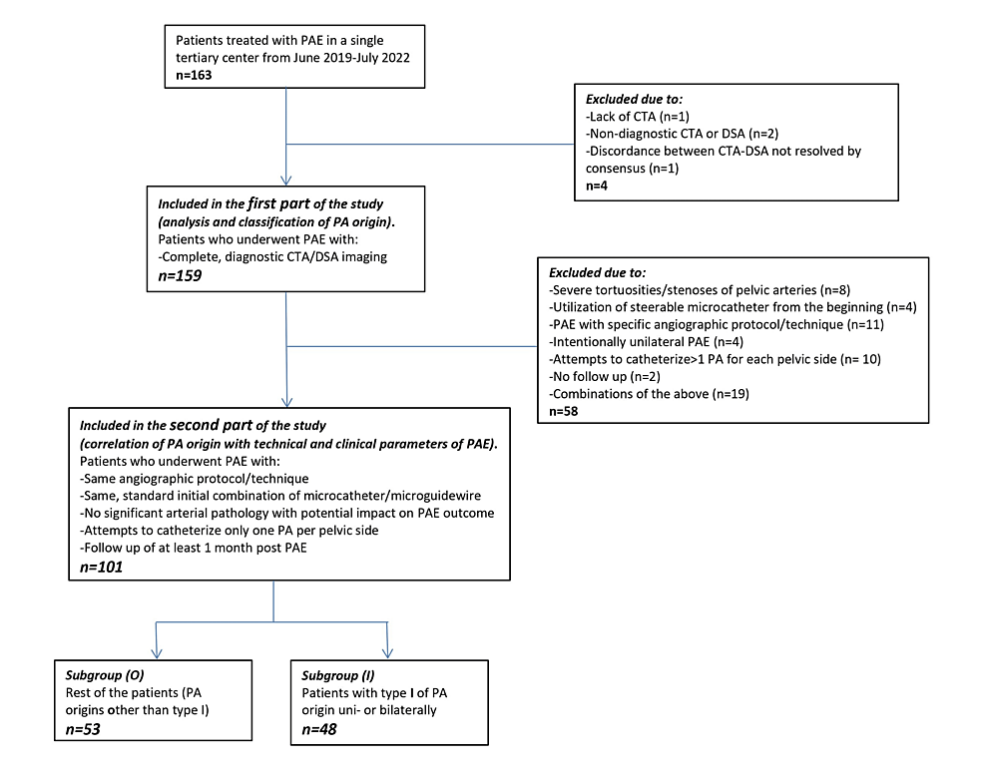
Flowchart of the study. PA: prostatic artery; PAE: prostatic artery embolization; DSA: digital subtraction angiography; CTA: computed tomographic angiography

Details regarding the patients who were included in the two parts of the study, as well as regarding the excluded patients, are provided in Figure 1.

A total of 159 patients were included in the first part of the study. Baseline demographic and clinical data are presented in Table 2.

**Table 2:** Baseline demographic, anatomic, and clinical data for patients (n = 159) of this study. SD: standard deviation; BMI: body mass index; PV: prostate volume; LUTS: lower urinary tract symptoms

Variable	Value
Age (years, mean ± SD)	72 ± 9.9
BMI (mean ± SD)	26.8 ± 2.9
PV (mL, mean ± SD)	85.9 ± 47.6
Indication for PAE (proportion of patients)	
Moderate LUTS	59/159
Severe LUTS	49/159
Chronic urinary retention	45/159
Hemorrhage of prostatic origin	6/159

In 123/159 (77.3%) patients, one PA was identified in each pelvic side. In 3/159 (1.9%) patients one PA was identified in one pelvic side, but no PA could be visualized contralaterally. In 28/159 (17.6%) patients, a double PA was found on one pelvic side, with a single PA on the other side. In 3/159 (1.9%) patients, bilateral duplication of PA was observed and in 2/159 (1.3%) patients a triple PA was found on one pelvic side with a single PA contralaterally. Overall, more than one PA per pelvic side was observed in 20.8% of the patients (11.3% of the studied pelvic sides).

**Figure 2:**
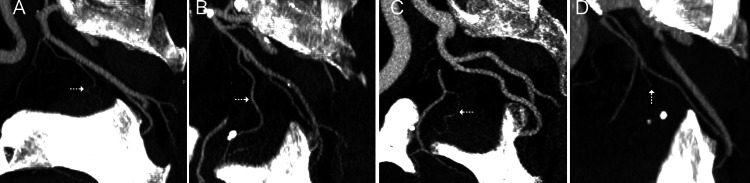
CTA/MIP images of the 4 main types of PA origin. (A) Type I, the common trunk of PA with the superior vesical artery. (B) Type II, PA origin from the anterior division of the internal iliac artery. (C) Type III, PA origin from the obturator artery. (D) Type IV, PA origin from the internal pudendal artery. PA: dotted arrow in all images. CTA: computed tomographic angiography; MIP: maximum intensity projection; PA: prostatic artery

From a total of 355 identified PAs, type I was represented by 110 (31%) arteries, type II by 58 (16.3%), type III by 45 (12,7%), type IV by 110 (31%), and type V by 32 arteries (9%) (Figure 2).

**Figure 3:**
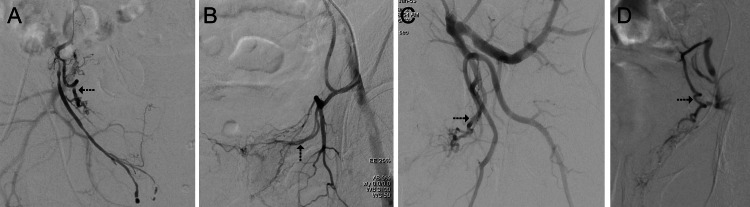
DSA images of less common PA origins found in this study. (A) PA origin from the accessory internal pudendal artery. (B) PA origin from the replaced obturator artery, which itself arises from the external iliac artery. (C) PA origin in the form of trifurcation from the anterior division of the internal iliac artery. (D) PA origin from the inferior gluteal artery. PA: dotted arrow in all images. DSA: digital subtraction angiography; PA: prostatic artery

The most common of type V origins was from the accessory IPA (21/32 or 5.9% of all the identified PAs). Less frequent subtypes of PA origin were (Figure 3): the common trunk of PA with two or more other arteries (in the form of tri, quadrifurcation, etc.) from the anterior division of IIA (5/32 cases). PA origin from the replaced obturator artery (which itself originated from the external iliac artery, 4/32 cases). PA origin from the inferior gluteal artery (2/32 cases).

Of the 123 patients with one identified PA on each pelvic side, symmetry in PA origin (same type of origin bilaterally) was observed in 51 (32%) patients, with 21, 4, 5, 20, and 1 patient(s) for bilateral types I, II, III, IV, and V, respectively.

From the studied population of the first part, a subgroup of 101 patients (202 pelvic sides, 202 PAs) were included in the second part of the study. Representation of the various types of PA origin in this subgroup was as follows: type I, 64/202 PAs; type II, 32/202 PAs; type 3, 27/202 PAs; type IV, 62/202 PAs; type V, 17/202 PAs. Type I was associated with significantly more incidences of difficult and failed super-selective catheterization compared to all other types together (27/64 vs. 18/138, p < 0.001) (Table 3).

**Table 3:** Outcomes of attempt for super-selective catheterization for different types of PA origin from the second part of the study. PA: prostatic artery

Type of PA origin	Outcome of attempt (numbers of cases)		
	Success	Difficulty	Failure
I	37	15	12
II	25	3	4
III	24	2	1
IV	56	3	3
V	15	2	0
Total	157	25	20

Types III, IV, and V exhibited comparatively low rates of difficult and failed super-selective catheterizations. The unfavorable angle of PA origin was by far the most frequent cause of difficult or failed PA catheterization (24/45 cases, 53.3%) and was more frequently associated with type I being the underlying cause of 24/27 (88.8%) incidences of failed or difficult type I catheterizations. Causes of failure or difficulty of catheterization per type of PA origin are provided in Table 4.

**Table 4:** Causes of failure or difficulty in catheterization of PA per type of PA origin. PA: prostatic artery

Type of PA	Causes of failure or difficulty in catheterization of PA (n of cases)				
	Unfavorable angle of PA origin (or of its common trunk)	PA origin at the very proximal part of the parent artery	Misidentification of the origin of PA or of the origin of its parent artery	Difficulty in the selection of PA from neighboring larger branches	Unclear
I	24				3
II	2		2	2	1
III		1	2		
IV	3	3			
V				2	
Total	29	4	4	4	4

To investigate differences in radiation exposure between different types of PA origin, patients of the second part of the study were further divided into two subgroups: Subgroup (I) with type I PA origin of at least one pelvic side (n = 48 patients, 32 with unilateral and 16 with bilateral type I origin), and subgroup (O), comprising the rest of the patients, with the other types of origin (n = 53). PAE of subgroup (I) was associated with significantly higher radiation exposure compared to subgroup (O), with FT of 47.7 vs. 41.1 minutes (p = 0.007) and DAP of 21,060 vs. 14,392 μGy^.^cm^2^ (p < 0.001), respectively. Of note, differences in age, prostate volume, and body mass index between subgroups (I) and (O) were not statistically significant (p = 0.19, p = 0.7, and p = 0.15, respectively).

Clinical success rates at 3, 6, 12, and 18 months post-PAE were 81.3%, 78.7%, 75.8%, and 75.8%, respectively, for subgroup (I) and 94.3%, 83.8%, 83.8%, and 83.8%, respectively, for subgroup (O). Differences between the two subgroups were not statistically significant (p = 0.137). No major complications were observed in the entire study population.

## Discussion

In the first part of this study, the type of PA origin was systematically investigated with CTA and DSA for the first time in a Greek population with BPH. Several studies from other countries have utilized the same system for the categorization of PA origin, and, not surprisingly, there is variance between the results of the present and previous studies [3-6,8-11], likely due to racial and ethnic differences in pelvic arterial anatomy. In the studied population, the prevalence of the two most common types (I and IV, 31% each) was lower and higher, respectively, compared to the majority of previous articles. Regarding types II and III, they are less common than types I and IV, both in the present and in the largest part of previous work, although their prevalence varies significantly among the reports (6-31.1% and 4.7-23.8%, respectively). Interestingly, the herein-reported prevalence of type V (9%) is significantly higher compared to all but one [6] of the previous studies. The same applies to the main representative of type V in this work, namely, PA origin from the accessory IPA. This artery served as the parent artery for 5.9% of the total number of the herein-studied PAs compared to only 0.6-2.1% elsewhere [2,3]. Relevant data from other countries are summarized in Table 5.

**Table 5:** Previous imaging studies investigating PA origin of populations from different countries. DSA: digital subtraction angiography, CBCT: cone-beam computed tomography, CTA: computed tomographic angiography

First author	Year	Country	Patients (n)	Imaging modality	Prevalence of each type of origin (%)				
					I	II	III	IV	V
Eldem et al. [8]	2021	Turkey	68	DSA	36.1	10.9	18.5	28.6	5.9
Shaker et al. [9]	2021	Egypt	210	DSA	39.9	11	19	29.2	0.9
Boeken et al. [10]	2020	France	215	CBCT, DSA	37.2	6	17	27.4	8
Enderlein et al. [2]	2019	Germany	104	CBCT, DSA	27.5	20	23.8	23.1	5.5
Schnappauf et al. [11]	2019	Germany	22	CBCT, DSA	36.3	27.2	15.9	20.4	0.2
Xuan et al. [6]	2019	Vietnam	330	DSA	33.9	10.4	18.3	23.5	13.9
Maclean et al. [5]	2017	United Kingdom	110	CTA, DSA	17.7	22.7	19.1	36.4	4.1
Wang et al. [4]	2017	China	148	CBCT, DSA	37.1	31.1	4.7	24.2	2.9
de Assis et al. [3]	2015	Brazil	143	DSA	28.7	14.7	18.9	31.1	5.6

More than one PA per pelvic side was also more frequently diagnosed in the present study (20.8% of all patients) compared to others (2.5-14.9%) [3,8-10]. In fact, a higher frequency of PA duplication has been recorded only in one previous study [1]. Careful investigation for supernumerary PAs is, therefore, required, both in the pre-interventional CTA and during PAE.

The results of the second part of the study revealed significant differences in the degree of technical difficulty associated with access to different PA origins. Type I proved the most technically challenging, with significantly more incidences of failed and difficult catheterization compared to all the other types together. Type I was also associated with increased radiation dose, most likely as a result of the prolonged FT and of the numerous DSA runs, required for clear visualization and catheterization of the common SVA-PA trunk and, then, of the PA. This is in line with previous studies [2,8,12], which (despite their different methodology) have found a significant correlation of type I with unilateral PAE (as a result of failed catheterization of the contralateral side) and with increased DAP, FT, and procedural time. Moreover, other researchers have found that, compared to others, type I required significantly more microcatheters for catheterization, with obvious implications in the cost of PAE [10]. Knowledge of the challenges associated with type I could facilitate preparations for PAE (regarding, for example, the availability of steerable microcatheters); PAE candidates with this PA anatomy should also be aware of the possibility of a prolonged procedure with increased radiation dose and suboptimal technical outcome. The present study also showed a slightly lower clinical success rate of PAE of type I, most likely as a result of the increased risk for unilateral (less effective) PAE associated with this type [12]. Although this difference in clinical success rates between type I and the other types failed to reach statistical significance, it probably merits further research due to its potential impact on prognosis and patient counseling.

On the other hand, regarding “technically easier” types of PA origin, the findings of the present study are also largely in line with previous work. Type III is considered probably the easiest type for PA catheterization because the obturator artery is almost always easy to catheterize and because of the favorable angle of PA origin from the obturator [2,8]. Moreover, types III and IV appear to be more suitable for the application of the ’’AP-PAE’’ approach, which is easier and associated with lower radiation exposure compared to standard PAE with oblique views [13].

There is a paucity of data regarding technical issues associated with type V [3,14,15]. Interestingly, this heterogeneous group of uncommon types of PA origin proved generally easy to catheterize. This probably highlights the value of accurate pre-procedural mapping with CTA.

This work has several limitations. It is a single-center, retrospective study and the number of patients is lower compared to that of some of the previous studies. Radiation data were not calculated separately for each pelvic side; as a result, it was not possible to study in detail the impact of each type on FT and DAP. Characterization of ‘’difficult’’ catheterizations was based exclusively on the need for additional dedicated devices; prolonged procedural times and maneuvers required in challenging cases were not taken into account. CBCT, which provides more detailed vascular imaging than CTA [16], was not routinely utilized for the study of PA origin. Finally, measurements of PA diameter were not included in the study.

## Conclusions

This is the first angiographic study of PA origin in Greece. It was demonstrated that types I and IV of PA origin were the most common and had the same prevalence (31% each). Supernumerary PAs and rare origins were detected more frequently than in many previous studies. Type I of PA origin showed significantly higher technical difficulty compared to the others, being associated with higher rates of difficult or failed catheterization and with higher radiation exposure. The negative effect of type I on the clinical outcome of PAE was not statistically significant, but probably merits further investigation.
